# *Proteus mirabilis* thoracic vertebral osteomyelitis: a case report

**DOI:** 10.1186/s13256-021-02890-7

**Published:** 2021-05-28

**Authors:** Ming-Hsiu Chiang, Mei-Hui Lee, Yung-Ching Liu, Chi-Hung Lee

**Affiliations:** 1 School of Medicine, College of Medicine, Taipei Medical University, No. 250, Wuxing St., Xinyi Dist., Taipei, 110 Taiwan; 2 Division of Infectious Diseases, Shuang-Ho Hospital, Taipei Medical University, No. 291, Zhongzheng Rd., Zhonghe Dist., New Taipei City, 235 Taiwan; 3 Department of Internal Medicine, School of Medicine, Taipei Medical University, Taipei, Taiwan

**Keywords:** *Proteus mirabilis*, Osteomyelitis, Thoracic vertebrae, Bacteremia, Case report

## Abstract

**Background:**

*Proteus mirabilis* is the second most common pathogen that causes urinary tract infections after *Escherichia coli*. In rare cases, it is associated with vertebral osteomyelitis. The underlying mechanism of this relationship may be related to the retrograde dissemination of bacteria through the paravertebral venous plexus.

**Case presentation:**

We report a case of an 80-year-old Taiwanese woman who had recurrent episodes of fever and chronic back pain for 1 year. All blood cultures were positive for *P. mirabilis*. Inflammation scans and magnetic resonance imaging revealed a previously undetected vertebral lesion between the seventh and eighth thoracic vertebra. She responded well to treatment with antibiotics, reporting considerable relief of back pain and no fever recurrence at the 4-month follow-up.

**Conclusions:**

Chronic back pain is a common but often dismissed symptom among the older population; osteomyelitis should be considered in patients with recurrent fever or neurological symptoms. Old age, chronic renal failure, and diabetes mellitus are possible predisposing factors for osteomyelitis. Our findings suggest that long-term treatment with antibiotics is effective for osteomyelitis caused by *P. mirabilis,*, although surgery is required for abscess formation or serious vertebral destruction.

## Background

Spinal osteomyelitis is usually a monomicrobial infection most commonly caused by *Staphylococcus aureus* [1]. In rare cases, spinal osteomyelitis can be caused by *Proteus mirabilis*; vertebral osteomyelitis caused by this organism has been reported in fewer than 30 individuals over the last 75 years [2]. The underlying mechanism of infection remains unclear. *P. mirabilis* is the second most common pathogen in urinary tract infections (UTIs). Therefore, some researchers have proposed a transmission pathway involving retrograde dissemination of the pathogen from the urinary tract to the vertebrae through the Batson plexus [3]. Research indicates that the Batson plexus, which connects the deep pelvic veins to the internal vertebral venous plexuses, may be responsible for bone metastases of pelvic organ cancers, such as prostate cancer [4].

Vertebral osteomyelitis often presents with varied symptoms and is therefore a diagnostic challenge for clinicians. In the present case report, we explain our clinical process for identifying the infection source and causative pathogen in the case of an older adult woman who had recurrent fevers. In addition to describing conservative treatment plans for *P. mirabilis* vertebral osteomyelitis, we comprehensively reviewed previous reports of similar cases and their respective diagnostic approaches.

## Case presentation

An 80-year-old Taiwanese woman was admitted to the emergency room of our hospital with intermittent fever and colic abdominal pain lasting for 3 days. She also expressed concerns about severe back pain lasting for over 1 year, and she had no prior trauma or history of surgery. She had type 2 diabetes mellitus and hypertension, which had been under medical control for over 10 years. She was [1] taking a calcium channel blocker once daily as an antihypertensive drug and [2] having an insulin pen injection twice daily for diabetes control. She also had hepatitis B infection, for which she was undergoing treatment with tenofovir, and mild dementia. The patient had no history of alcohol consumption or smoking. Her husband had died several years previously, and she had two sons and one daughter. Before retiring, she worked as a deputy section manager in a local farm alliance. In the emergency department, she had a fever (body temperature, 38.2 ℃), sinus tachycardia (heart rate, 107 beats/minute), and high blood pressure (146/84 mmHg). After admission to ward, her body temperature returned to normal (36.7 ℃), but her sinus tachycardia (108 beats/minute) and high blood pressure (136/84 mmHg) persisted. We noted bilateral proximal weakness of the lower limbs without urinary sphincter involvement. According to the patient’s caregiver, this weakness symptom had progressed over the last few years. The patient could walk short distances but was dependent on a wheelchair most of the time. Physical examination and sonography ruled out pyelonephritis and hydronephrosis. The patient’s other neurological and physical examinations reveal no abnormalities. This was the patient’s third admission to our hospital; she had been admitted twice in the previous 6 months for intermittent fever. Mid-portion urine was collected during each admission, and routine urine analysis revealed pyuria, positive urine nitrates, and bacteriuria. Urine aerobic culture showed mixed growth of *P. mirabilis* and *Escherichia coli* during the first hospital stay and *E. coli* alone during the second admission. Three sets of aerobic blood cultures were taken from the patient at both admissions, and the results indicated gram negative bacilli later identified as *P. mirabilis* bacteremia. Anaerobic blood culture was also conducted during her second hospital stay, and the results were negative. Laboratory examination results during her first and second admissions (written on the left and right, respectively) were as follows: white blood cell count, 8.3 × 10^3^/µL and 11.0 × 10^3^/µL; C-reactive protein (CRP) level, 5.1 mg/dL and 6.8 mg/dL; neutrophil percentage, 80.6% and 68.3%; blood urea nitrogen, 13 mg/dL and 20 mg/dL; and serum creatinine value, 0.8 mg/dL and 0.98 mg/dL, respectively. Flomoxef was prescribed, and fever subsided within 1 day in her previous admissions. During her third admission, laboratory tests also revealed leukocytosis (white blood cell count, 8.2 × 10^3^/µL), high CRP level (5.2 mg/dL), normal neutrophil percentage (77.4%), and pyuria combined with positive urine nitrate and bacteriuria. Urine culture revealed *E. coli* growth > 10^4^ CFU/mL, and no specific indications were obtained from abdominal sonography, chest X-ray, or computed tomography findings.

**Fig. 1:**
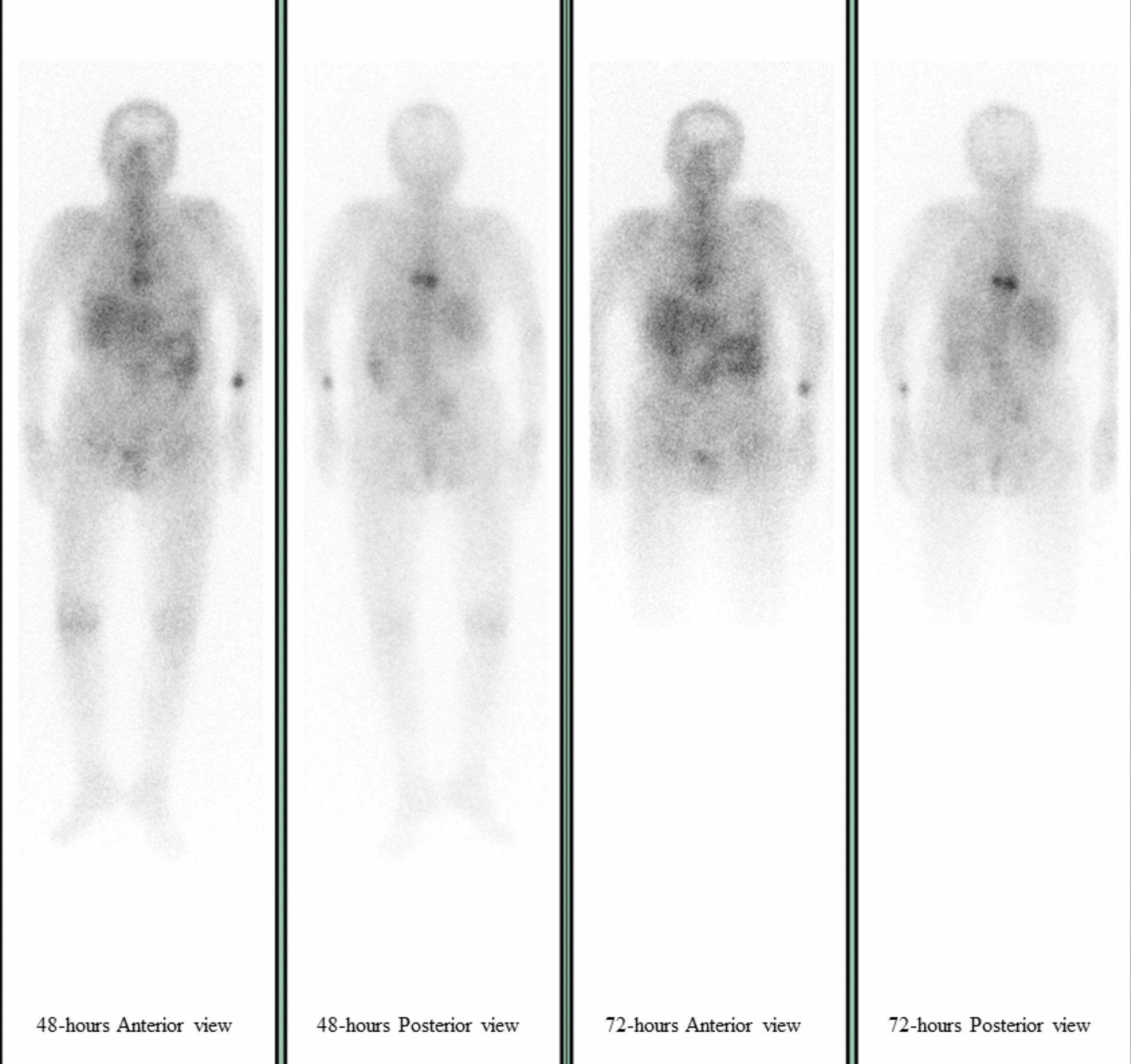
Gallium scan of the patient showing increased Ga67 citrate uptake in the ninth thoracic vertebra area

**Fig. 2:**
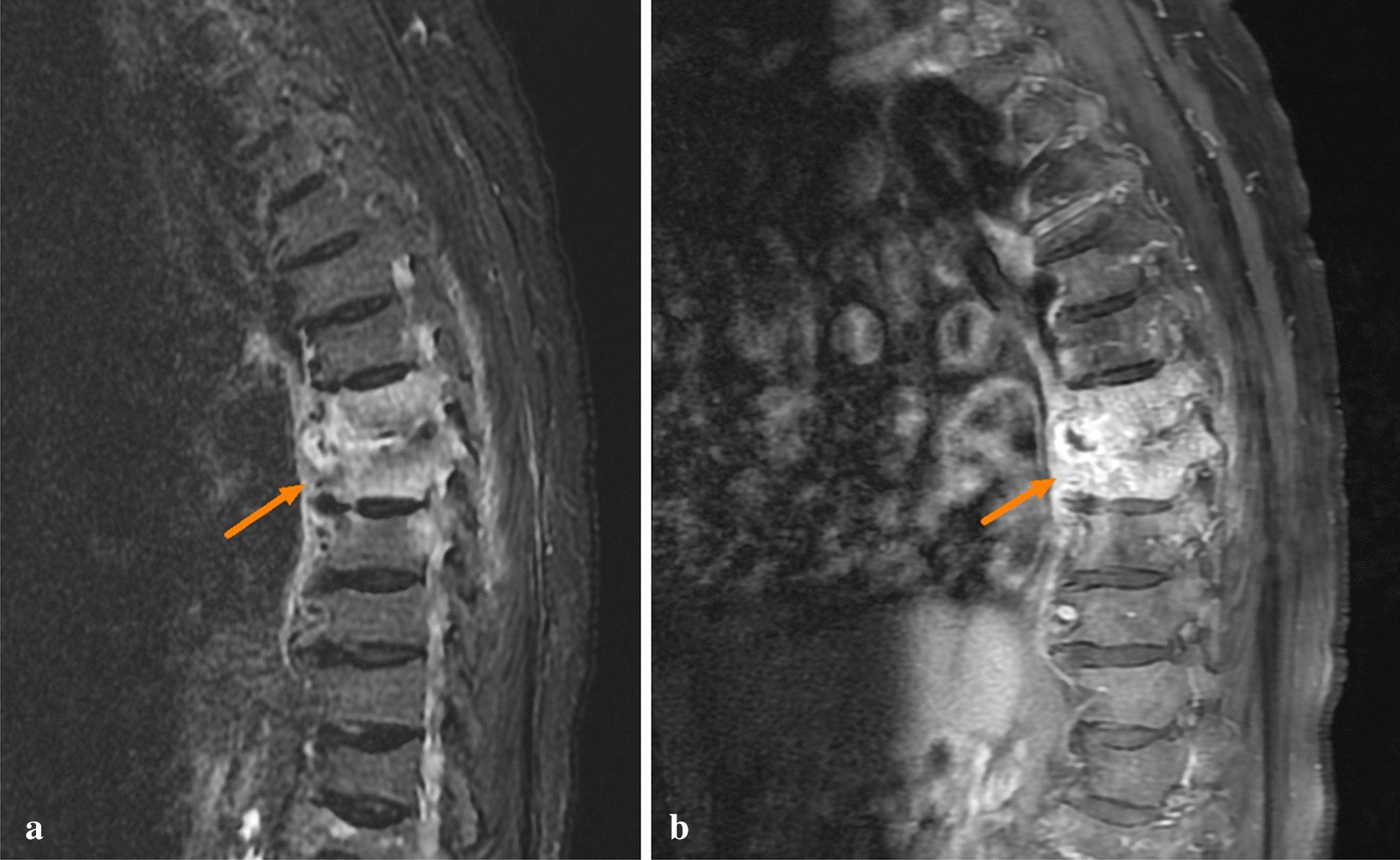
Magnetic resonance imaging of short T1 inversion recovery sequence (**a**) and contrast-enhanced T1 sequence (**b**). Enhanced signals between the seventh and eighth thoracic vertebra segments (arrows )

After starting flomoxef empirical treatment on day 1 of admission, the patient’s fever subsided promptly and her abdominal pain was also alleviated. Thus, we downgraded her regimen of antibiotics from flomoxef to cefuroxime on the following day. The patient’s fever did not recur during the remainder of her hospital stay. Although the urine culture indicated a possible UTI, a gallium scan was also conducted to rule out other potential causes of recurrent fever and to determine the infection source of *P. mirabilis* bacteremia. The scan revealed increased Ga67 citrate uptake in the ninth thoracic vertebra, which aligned with the location of the patient’s back pain (Fig. 1). We also conducted magnetic resonance imaging (MRI) several days later. Both the short T1 inversion recovery (STIR) sequence image (Fig. 2a) and the contrast-enhanced T1 sequence (Fig. 2b) depicted enhanced signals between the seventh and eighth thoracic vertebral segments, which indicated infectious spondylodiscitis. In addition, mild spinal canal stenosis was noted in the same area, which may have caused the patient’s neurological symptoms. We consulted orthopedic specialists, and they suggested conservative treatment. The patient’s family was also opposed to surgery. We did not assess whether the patient had tuberculosis (TB) infection; however, the possibility of TB infection was low because the patient reported no TB-associated symptoms in the past decade, and no TB-like lesions were identified in her chest X-rays.

Intravenous cefuroxime injections during the entire course of hospitalization stabilized the patient’s clinical condition, and white blood cell count was normal during hospitalization. Because of the considerable improvement in her condition, she was discharged on the 19th day of hospitalization with oral cefuroxime as her take-home medication. She reported to the outpatient department for follow-up once a month for 4 months. Her erythrocyte sedimentation rate (ESR) had decreased from 91 mm/hour to approximately 30 mm/hour by her fourth outpatient department visit. After the initial 4-month follow-up period, the patient came to the outpatient department once every 3 months. At her last follow-up, which was approximately 10 months after discharge, the patient had no complaints of back pain or fever. According to the patient and her caregivers, she became more energetic and was no longer experiencing proximal weakness of the lower limbs.

## Discussion and conclusions

In this case report, an elderly woman with recurrent fever was admitted to the hospital and eventually diagnosed with *P. mirabilis* vertebral osteomyelitis. She had a clinical history of two previous *P. mirabilis* infections and bacteremia episodes. In her previous hospital admissions, her fever resolved within 1 day after starting antibiotics but resurged after termination of antibiotic treatment, a perplexing clinical presentation that has not been reported in previous *P. mirabilis* vertebral osteomyelitis cases. However, the patient also exhibited continuous UTI, progressive back pain, and deteriorating neurological functions, with the latter two being well-described clinical signs of *P. mirabilis* vertebral osteomyelitis in the literature.

Vertebral osteomyelitis is an infectious disease that affects the spine. It is frequently misdiagnosed because of its insidious onset and unclear clinical course. Older adults are particularly vulnerable to this disease. Its incidence is approximately 2.2–5.8 per 100,000 person years [1]. The most common symptoms of vertebral osteomyelitis are back pain, fever, and local tenderness. Severe cases involve neurologic impairments, such as sensory loss, weakness, and radiculopathy, due to bone destruction and subsequent spinal cord compression [5]. Other means of diagnosis include laboratory findings of increased ESR, CRP, and leukocytosis. MRI is the most sensitive mode of examination for detecting vertebral osteomyelitis, and its diagnostic accuracy can be maximized if it is performed using contrast agents, fat-suppressed sequences, and subtraction images to prevent bone marrow and fat tissue from emerging in T1-weighted sequences [6].

Vertebral osteomyelitis caused by *P. mirabilis* is rare. A retrospective cohort study reported that, among 253 episodes of vertebral osteomyelitis, only 5 cases were secondary to *P. mirabilis* infections [7]. The first case of vertebral osteomyelitis caused by *P. mirabilis* was published in 1934, in which the patient was infected after undergoing cystoscopy [8]. In the subsequent years, several case reports of vertebral osteomyelitis caused by *P. mirabilis* infection have been published [2, 3, 9–13]. Diagnoses have mostly been based on blood or urine culture results combined with radiological evidence, and some studies have confirmed *P. mirabilis* infection through direct biopsy and pus culture. The majority of patients have recovered completely after long-term treatment with antibiotics. The regimens and treatment durations have varied across studies; patients have been prescribed different antibiotics, including quinolone, cephalosporin, and aminoglycoside, with treatment periods lasting from 5 weeks to over 3 months. Surgery is occasionally necessary, particularly in cases involving abscesses formation or severe bone destruction. Immediate drainage of abscess, disc excision, and internal vertebral stabilization have been performed in these patients [3, 12].

Our patient initially presented with recurrent fever and *P. mirabilis* bacteremia, which was confirmed by three sets of positive *P. mirabilis* blood culture results in both of her previous admissions. We initially suspected a UTI to be the source of infection based on the patient’s quick response to antibiotic treatment. However, her fever recurred soon after discharge. This was an atypical clinical presentation of UTI. In addition, her persistent back pain and bilateral lower limbs weakness prompted us to consider the possibility of an undetected infectious source of bacteremia, such as an infective vertebral disease. Consequently, we investigated the matter more thoroughly. Vertebral osteomyelitis was detected through inflammation scans and MRI images. Although TB-induced vertebral osteomyelitis could not be ruled out, we judged that TB infection was not likely considering the patient’s past history, chest X-rays, and significant improvements with cefuroxime treatment. We did not perform surgical aspiration of the lesion because of opposition from the patient’s family; however, considering that *P. mirabilis* was the only pathogen detected in the blood cultures, vertebral osteomyelitis caused by *P. mirabilis* is the most likely final diagnosis.

## Conclusions

Because chronic back pain in the older adult population is often dismissed as a common ailment, the diagnosis of spondylodiscitis requires a high index of suspicion. Physicians should consider the possibility of undiscovered osteomyelitis when the patient presents with concurrent neurologic symptoms and bacteremia. Once TB osteomyelitis is ruled out, pyogenic osteomyelitis of other pathogens should be considered. Vertebral osteomyelitis caused by *P. mirabilis* is extremely rare, and old age, chronic renal failure, and diabetes mellitus are possible predisposing factors. Previous case reports have also indicated recurrent UTI episodes despite sufficient antibiotic treatment; this symptom, along with the presence of permanent indwelling urinary catheter, should alert clinicians to the possibility of causative pathogenic organisms other than *S. aureus* [2]. Vertebral osteomyelitis caused by *P. mirabilis* can be sufficiently treated with long-term antibiotics administrations; the duration of treatment was around 4 months in the present case, with intravenous injection for the first 3 weeks and oral admission for the remainder of treatment. Overall, the disease has a favorable prognosis.

## Data Availability

All data generated or analyzed during this study are included in this published article.

## Abbreviations

CRP: C-reactive protein
ESR: Erythrocyte sedimentation rate
MRI: Magnetic resonance imaging
UTI: Urinary tract infections
TB: Tuberculosis
